# Protective Effect of Rutin and Naringin on Sperm Quality in Streptozotocin (STZ) Induced Type 1 Diabetic Rats

**Published:** 2011

**Authors:** Raju Butchi Akondi, Phani Kumar, Akula Annapurna, Manasa Pujari

**Affiliations:** *Department of Pharmacology, College of Pharmaceutical Sciences, Andhra University, Visakhapatnam, A.P, 530003, India.*

**Keywords:** Type 1 diabetes, Oxidative stress, Male infertility, Rutin, Naringin

## Abstract

Oxidative stress is one of the important causes of the type 1 diabetes induced changes in the sperm quality. Bioflavonoids, Rutin 10 mg/Kg and Naringin 10 mg/Kg were evaluated for their protective effects on sperm parameters, oxidative stress, and histopathology of type 1 diabetic rats. Results demonstrated the reduction in sperm count, sperm motility and vitality in diabetic rats. Mass drug administration (MDA) levels were increased and superoxide dismutase (SOD) catalase levels were decreased. Histopathological changes were evident and in accordance with the above results. In the treatment groups, both Rutin and Naringin in combination with insulin treatment in diabetic rats produced protection from diabetes and improved all the sperm parameters, decreased the MDA levels and increased the SOD and catalase levels. Protection was evident in histological examination. Our data suggests that the possible protection of testicular tissue and reproduction from oxidative stress have been induced by type 1 diabetes mellitus.

## Introduction

Diabetes mellitus (DM) is a chronic metabolic disorder characterized by hyperglycemia caused by abnormal insulin production, insulin resistance or often both (1). Type 1 DM is also known as juvenile onset diabetes which occurs in young age. Diabetic patients generally experience sexual abnormalities like sexual dysfunction, impotence and infertility (2).

Various experiments conducted on streptozotocin (STZ) induced diabetic rats supported the relation between male infertility and diabetes mellitus (1, 2). It is indicated that the oxidative stress is more in hyperglycemia state, due to the excess production of reactive oxygen species (ROS) and decreased efficiency of anti-oxidant enzyme defenses (3). Several studies reported that diabetes mellitus induced hyperglycemia impairs male fertility by altering cell function of testes in animal models (5-13).

Hyperglycemia associated biochemical changes leads to oxidative stress and vascular effects. The generation of reactive oxygen species (ROS) is mainly due to glucose autoxidation, activation of polyol pathway, increased glycolysis, PKC activation (protein kinase C) and hexosamine pathway (1, 2). Increased reactive oxygen species (ROS) cause the oxidation of proteins, lipids and damage macro molecules like DNA (14-16).

Excessive oxidative stress impairs male fertility by altering the cell function *i.e. *sperm motility (17), DNA damage by gene mutations, denaturation and DNA base pair oxidation (14). DNA fragmentation (15, 16) is associated with declining motility, count, viability and diminished fertility of the human sperm (3).

Cellular antioxidant defenses are classified into primary antioxidant enzymes which include superoxide dismutase (SOD), Catalase (CAT) and glutathione peroxidase (GPX), secondary non-enzymatic antioxidants such as ubiquinol, vitamin E, vitamin C and *b *carotene. SOD converts superoxide radical to hydrogen peroxide, catalase enzyme catalyzes the decomposition of hydrogen peroxide to water (H_2_O) and oxygen molecule (O_2_) where as glutathione peroxidase catalyzes the reduction of hydrogen peroxide and organic hydroperoxide into water and corresponding alcohol at the expense of Glutathione (GSH). The stability and capacity of the antioxidant defense against ROS during chronic diabetes plays an important role in an outcome of long term complications caused by ROS (18). 

Antioxidants may be useful in the treatment of male infertility (11). In 2008, Hulya Aybek *et al*., reported beneficial effects of vitamin E on sperm parameters in type I diabetic rats. Bioflavonoids are often referred as classical antioxidants. Bioflavonoids like rutin and Naringin were proved as antioxidants. Bioflavonoids have also shown simulative effects on sperm parameters. (19).

In the present study, we selected streptozotocin (STZ) induced diabetic rats as a model for type I diabetes mellitus and evaluated the effects of Rutin and Naringin on sperm parameters and oxidative stress in type I diabetic male rats. In this study, we estimated the sperm count, sperm motility, sperm viability, lipid peroxidation, and superoxide dismutase and also the catalase concentrations in streptozotocin-induced type I diabetic rats. To the best of our knowledge, this is the first study to evaluate the effects of Rutin and Naringin on testicular oxidative stress in type 1 diabetic rats.

## Experimental

Rutin, Naringin and streptozotocin were purchased from Sigma chemicals Ltd (Sigma, USA). Thiopental sodium injection was purchased from Neon Laboratories Ltd (Neon, MUMBAI). All other chemicals and reagents used were of analytical grade.

*Animals used in study*

The 4-month-old young male albino Wistar rats purchased from National Institute of Nutrition (Hyderabad, India), weighing 175-200 g were used in the study. Animals were maintained under the standard laboratory conditions at 25 ± 2°C, relative humidity of 50 ± 15% and normal photoperiod (12 h dark/ 12 h light). Commercial pellet diet (Rayons biotechnologies Pvt Ltd, India) and water were provided *ad libitum. *The experimental protocol has been approved by the institutional animal ethics committee. (Regd. No. 516/01/A/CPCSEA).

*Study design*

The rats were randomly divided to eight groups each containing of six. The groups were treated according to experimental protocol for duration of 45 days. These groups were as follows: Group 1: normal control. Group 2: diabetic control. Group 3: vehicle control; diabetic rats treated with 0.1% sodium carboxymethylcellulose (sodium cmc); Rutin and Naringin were dissolved in 0.1% sodium cmc and administered intra-peritoneally (IP). Group 4: diabetic animals treated with insulin SC 3 U/100 g body weight per day. Group 5: diabetic animals treated with both insulin and rutin, 3 U/100 g body weight per day and 10 mg/kg per day, respectively. Group 6: diabetic animals treated with rutin 10 mg/kg per day alone. Group 7: diabetic animals treated with both insulin and Naringin, 3 U/100 g body weight per day and 10 mg/kg per day, respectively. Group 8: diabetic animals treated with Naringin10 mg/kg per day alone. 

*Induction of diabetes*

Diabetes was induced by a single Intravascular/Intravenous (IV) injection of STZ, 45 mg/Kg of body weight, dissolved in citrate buffer (pH 4.5), into the tail vein of animals lightly anaesthetized with ether. Diabetes was confirmed after the third day of STZ injection by estimation of serum glucose using an auto analyzer (Screen master 3000)

*Surgical procedure*

On the 46^th^ day, animals were sacrificed with lethal ether anesthesia and laporatomy was conducted. Testes and epididymis were collected. The epididymis was used for the evaluation of sperm parameters. The right testis was processed for histopathological studies and the left one was homogenated for biochemical estimations. 

*Biochemical parameters estimation*

Malanoldehyde (MDA) levels in the testicular tissue were measured by the method developed by Ohkawa *et al*. (20). This is based on the measurement of absorbance of thiobarbituric acid malanoldehyde. The tissue MDA levels were expressed as nmol/g tissue. Super oxide dismutase (SOD) activity was determined by the method developed by Fridovich. (21) This method was based on the inhibition of superoxide radicals’ reaction with phenyl tetrazolium chloride. The specific activity was expressed in terms of units for milligrams of protein. Catalase activity was measured based on the Aebi method (22). The activity of catalase was based on the disappearance of hydrogen peroxide. It was expressed as μM of H_2_O_2_ metabolized/mg protein/min. One unit was defined as 1 pmol of H_2_O_2_ consumed per min and the specific activity was reported as units per milligram of protein. Protein was estimated by the method developed by Lowry (23).

*Collection of spermatozoa for evaluation of sperm count, sperm motility and sperm viability*

Epididymal spermatozoa were collected by cutting the cauda region of the epididymis into small pieces in 2 mL of normal saline pre-warmed to 37°C. Sperm was forced out of the cauda epididymis with fine forceps by putting pressure on lower region of cauda epididymis, not forcing out excess material *i.e*. immature cells. In this study, sperm motility, count, and viability were evaluated by using conventional methods (24-26). Progressive sperm motility was done immediately after the collection of sperms. The number of motile spermatozoa was calculated per unit area and expressed as sperm motility percentage. Sperm counts were done using hemocytometer and the results were expressed as millions/mL of suspension. Sperm viability was done using Eosin and Nigrosin stain. The dead sperm took up the stain. Hundred sperm cells were counted in order to obtain the percentage of live/death ratio.

*Histopathological examination*

The testis were fixed in 10% formalin and embedded in paraffin. Five-micron thick sections were prepared and stained with hematoxylin and eosin (H and E). The tissue sections were evaluated under light microscopy by a blinded pathologist.

*Statistical analysis*

The results are expressed as mean ± SD. Differences in tissue lipid peroxide levels, SOD and CAT were determined by factorial one-way analysis of variance. Individual groups were compared using Tukey’s test. Differences with p < 0.001 were considered statistically significant. Statistical analysis was performed using Graph Pad Prism software (Version 5).

## Results and Discussion

*Effect of Rutin and Naringin on fasting blood glucose levels*

Streptozotocin-induced diabetic rats had shown significant increase in blood glucose level compared to the normal control animals. The fasting blood glucose levels are presented in Table 1. 

**Table 1:** Effect of insulin, Rutin and Naringin on fasting blood glucose levels (mg/dL) of diabetic rats

**Groups**	**Sperm motility (%)**	**Sperm count (10** ^6^ **/mL)**	**Sperm viability (%)**
Control	61.83 ± 50.83	28.91 ± 2.48	74.66 ± 0.95
Diabetic	18.16 ± 0.48 *^(a)^	5.83 ± 1.17*^(a)^	25.16 ± 0.6*^(a)^
Diabetic + 0.1% Sodium CMC	18.83 ± 0.98*^(b)^	6.46 ± 1.08*^(b)^	23.83 ± 1.25*^(b)^
Diabetic + Insulin	41.66 ± 0.56 *^(c)^	15.67 ± 1.5*^(c)^	53.33 ± 0.71*^(c)^
Diabetic + Insulin + Rutin	58.16 ± 0.6 *^(d)^	26.16 ± 0.64*^(d)^	67.33 ± 0.88*^(d)^
Diabetic + Rutin	25.17 ± 3.48*^(f)^	10.33 ± 2.8	29.5 ± 2.85
Diabetic + Insulin + Naringin	50.83 ± 1.68*^(e), ^*^(h)^	21.170.83 *^(e)^, *^(h)^	59.17 ± 0.75 *^(e)^, *^(h)^
Diabetic + Naringin	20.67 ± 3.45	9.17 ± 3.75	28.17 ± 2.06

**Figure1:**
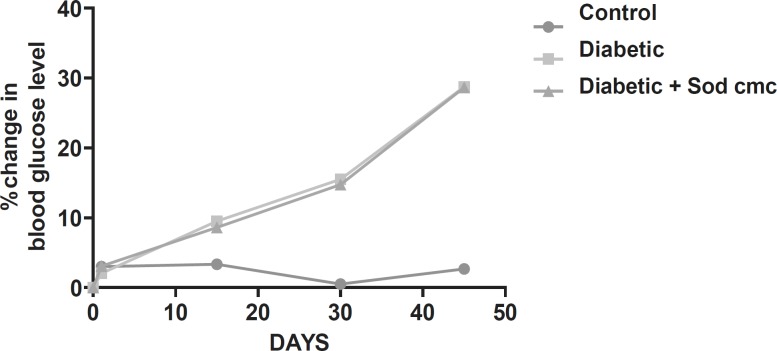
Graph showing effect of sodium CMC (vehicle) on fasting blood glucose levels of diabetic rats

**Figure 2:**
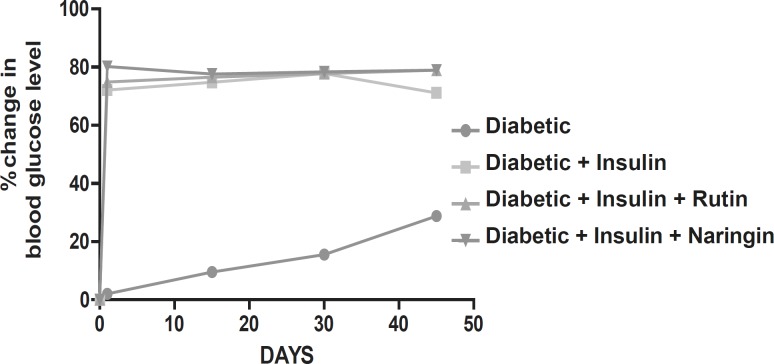
Graph showing effect of insulin, Rutin and Naringin on fasting blood glucose levels of diabetic rats

Diabetic animals treated with insulin have almost normal blood glucose levels. Diabetic animals treated with Rutin alone and Naringin alone and in combination along with insulin had no effect on blood glucose levels of streptozotocin induced diabetic rats. (Figures 1 and 2).

*Effect of Rutin and Naringin on sperm parameters of diabetic rats*

Sperm count, sperm motility percentage and sperm vitality percentage were significantly (p < 0.001) decreased in diabetic control group in comparison with sham control group. Values of sperm count, motility percentage and sperm vitality percentage were given in Table 2. 

**Table 2:** Effect of insulin, Rutin and Naringin on sperm parameters of diabetic rats.

**Groups**	**0** ^th^ **day**	**45** ^th^ **day**	**Change%**
Control	99 ± 1.0	96.33 ± 0.99	2.69
Diabetic	317 ± 3.56	408.17 ± 6.48	28.76
Diabetic + 0.1% Sodium CMC	319.33 ± 3.33	410.83 ± 4.99	28.64
Diabetic + Insulin	451.16 ± 2.77	103.17 ± 2.02	71.13
Diabetic + Insulin + Rutin	473 ± 2.41	99.17 ± 1.54	78.92
Diabetic + Rutin	317 ± 3.56	393.33 ± 3.18	24.07
Diabetic + Insulin + Naringin	474 ± 5.52	99.833 ± 2.06	78.93
Diabetic + Naringin	321.1 ± 2.60	399.17 ± 3.74	24.31
Data represents the means ± SEM of six animals per group* p < 0.01, compared as below			
Control vs. Diabetic		a	
Control vs. diabetic + Sodium CMC (diabetic control)		b	
Diabetic control vs. diabetic + insulin		c	
Diabetic + Insulin vs. diabetic + Insulin + Rutin		d	
Diabetic + Insulin vs. diabetic + Insulin + Naringin		e	
Diabetic control vs. Diabetic + Rutin		f	
Diabetic control vs. Diabetic + Naringin		g (NS)	
Diabetic + Insulin + Rutin vs. Diabetic + Insulin + Naringin		h	

**Figure 3:**
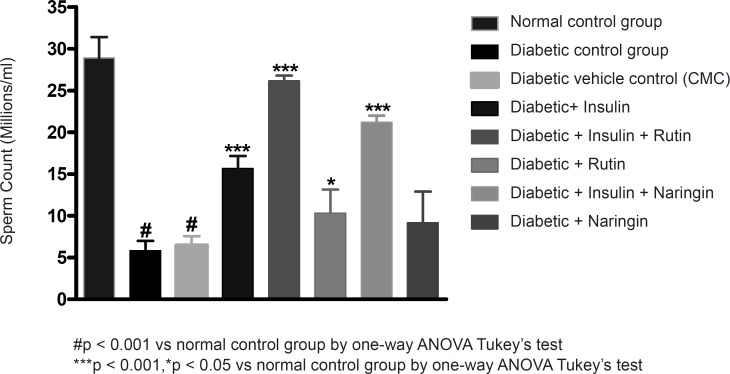
Effect of insulin, Rutin and Naringin on sperm count of diabetic rats

**Figure 4:**
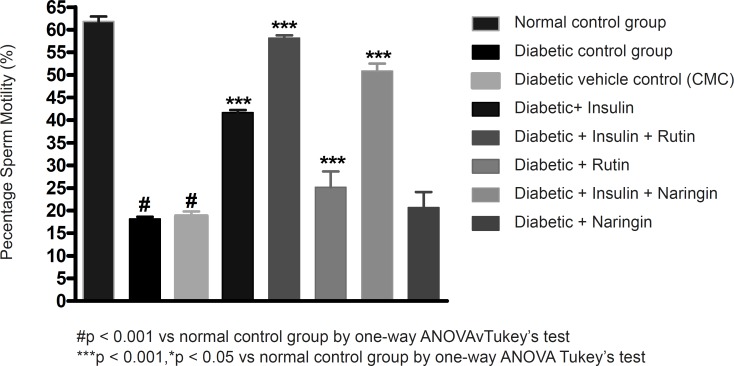
Effect of insulin, Rutin and Naringin on sperm motility of diabetic rats

**Figure 5:**
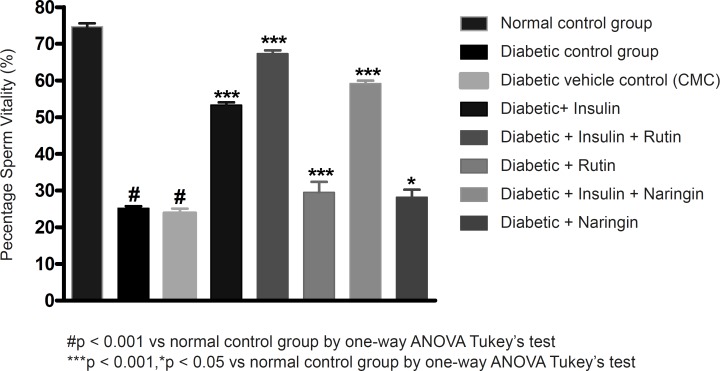
Effect of insulin, Rutin and Naringin on sperm vitality of diabetic rats

Insulin treatment has significantly (p < 0.001) increased sperm count, motility and vitality in diabetic rats. Insulin treatment with Rutin has further significantly (p < 0.001) increased the above sperm parameters. Rutin alone also shown significant (p < 0.001) increase in all the sperm parameters in diabetic rats, but insulin treatment alone has produced better efficacy. Interestingly, insulin in combination with rutin has shown much better efficacy than individual treatments of insulin and rutin. Similarly, insulin treatment with Naringin has also shown significant (p < 0.001) improvement in the sperm parameters. Naringin alone has not shown any significant improvement (Figures 3-4 and 5).

*Effect of Rutin and Naringin on oxidative stress and antioxidant status in diabetic rats*

Malondialdehyde is the end product of lipid peroxidation. The reaction of lipid peroxides with TBA has been widely adopted as a sensitive assay method for lipid peroxidation. MDA levels have significantly (p < 0.001) increased in diabetic control group compared to normal control group. Values of MDA were given in Table 3. All the three MDA levels of insulin, Rutin and Naringin, significantly decreased when given alone to diabetic rats. But insulin has shown better efficacy in comparison with Rutin and Naringin. Insulin treatment with rutin and insulin treatment with Naringin has further significantly (p < 0.001) decreased the MDA levels in diabetic rats. Interestingly, Insulin in combination with rutin has shown much better efficacy than all other groups.

**Table 3:** Effect of insulin, Rutin and Naringin on biochemical parameters of diabetic rats

**Groups**	**MDA** **(nmol/g tissue)**	**SOD** **(u/mg protein)**	**Catalase** **(u/mg protein)**
Control	190.75 ± 3.78	1755.52 ± 7.91	25.17 ± 1.25
Diabetic	380.32 ± 5.04*^(a)^	606.27 ± 5.70*^(a)^	7.83 ± 0.44*^(a)^
Diabetic + 0.1% Sodium CMC	382.75 ± 7.31*^(b)^	577.68 ± 25..21*^(b)^	8.50 ± 0.43*^(b)^
Diabetic + Insulin	274.57 ± 5.59*^(c)^	1020.53 ± 19.55*^(c)^	15.67 ± 0.77*^(c)^
Diabetic + Insulin + Rutin	225.28 ± 3.29*^(d)^	1540.12 ± 25.57*^(d)^	21.17 ± 0.64*^(d)^
Diabetic + Rutin	304.88 ± 3.26 *^(f)^	927.45 ± 18.41*^(f)^	11.5 ± 0.70*^(f)^
Diabetic + Insulin + Naringin	238.15 ± 3.60*^(e)^	1481.95 ± 11.14*^(e)^	19.83 ± 0.65*^(e)^
Diabetic + Naringin	329.3 ± 4.75 *^(g)^	811.76 ± 4.54*^(g)^	10.66 ± 0.88
Data represents the means ± SEM of six animals per group* p < 0.01, compared as below			
Control vs. Diabetic		a	
Control vs. diabetic + Sodium CMC (diabetic control)		b	
Diabetic control vs. diabetic + insulin		c	
Diabetic + Insulin vs. diabetic + Insulin + Rutin		d	
Diabetic + Insulin vs. diabetic + Insulin + Naringin		e	
Diabetic control vs. Diabetic + Rutin		f	
Diabetic control vs. Diabetic + Naringin		g	
Diabetic + Insulin + Rutin vs. Diabetic + Insulin + Naringin		h (NS)	

**Figure 6:**
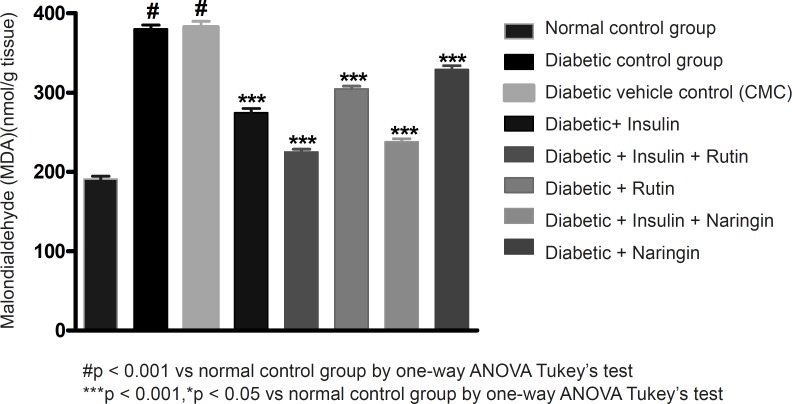
Effect of insulin, Rutin and Naringin on Malondialdehyde (MDA) levels of diabetic rats.

**Figure 7:**
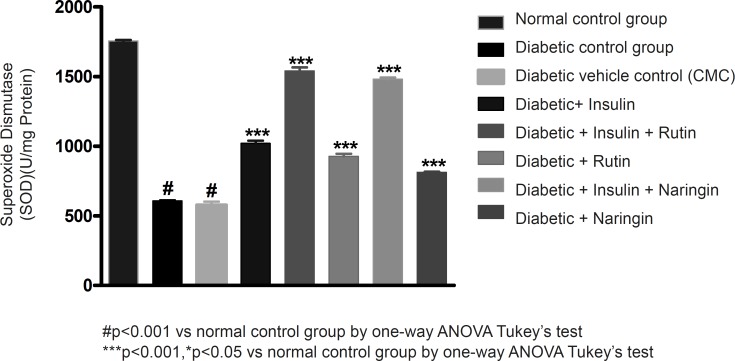
Effect of insulin, Rutin and Naringin on sod levels of diabetic rats

**Figure 8:**
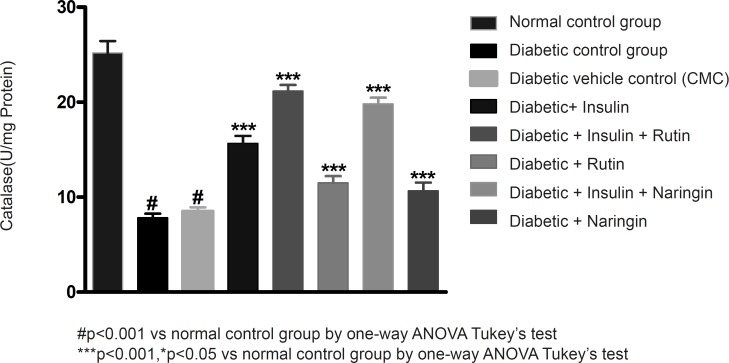
Effect of insulin, Rutin and Naringin on catalasel evels of diabeticrat s

In diabetic control group animals, the levels of endogenous antioxidant enzymes such as SOD and CAT in testicular tissue were significantly (p < 0.001) reduced compared to normal control group animals. Values of SOD and catalase were given in Table 3. Insulin treatment has significantly (p < 0.001) increased the SOD and catalase levels in diabetic rats. Insulin treatment with rutin and insulin treatment with Naringin has further significantly (p < 0.001) increased the SOD and catalase levels in diabetic rats. Insulin alone, rutin alone and Naringin alone also significantly (p < 0.001) increased the SOD and catalase levels in diabetic rats. But Insulin treatment alone has produced better efficacy than Rutin and Naringin. Interestingly, Insulin in combination with rutin has shown much better efficacy than individual treatments of insulin and rutin (Figures 6-7 and 8). 

*Effect of Rutin and Naringin on histology of diabetic rat testis*

**Figure 9:**
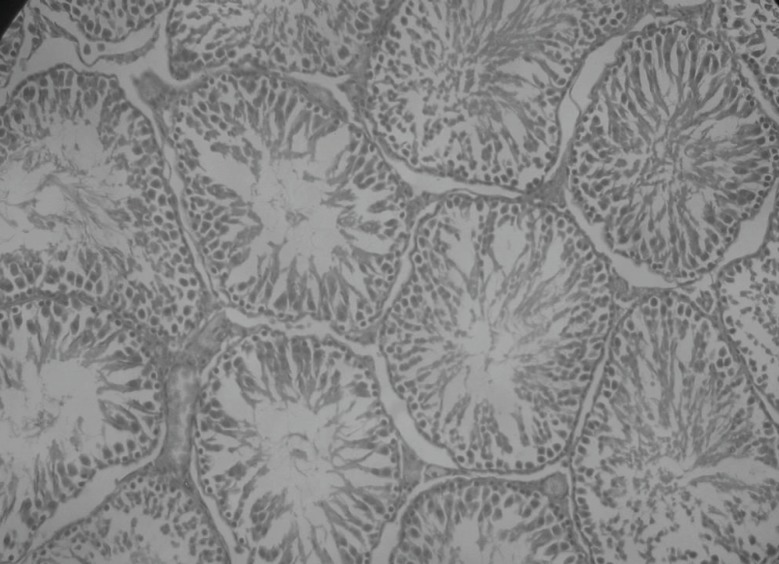
Histological picture of normal testis

**Figure 10.**
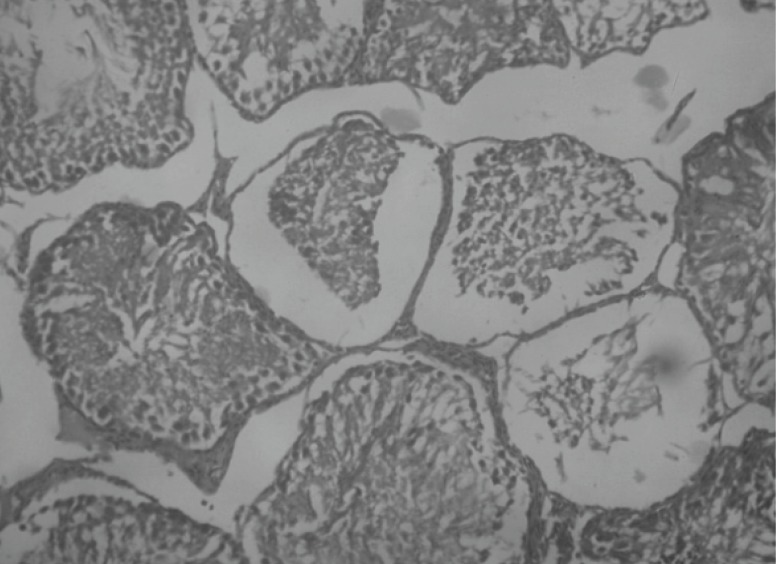
Histological picture of diabetic control rats testis

Histopathological examination of sections of diabetic rats’ testes showed lesions on seminiferous tubules and destruction of sperm cell with complete destruction of spermatogenic cells characterized by loss of embryonic cell and germ cell detachment (Figure 10). These were however, absent in the normal control rats which had intact seminiferous tubules (Figure 9).

**Figure 11:**
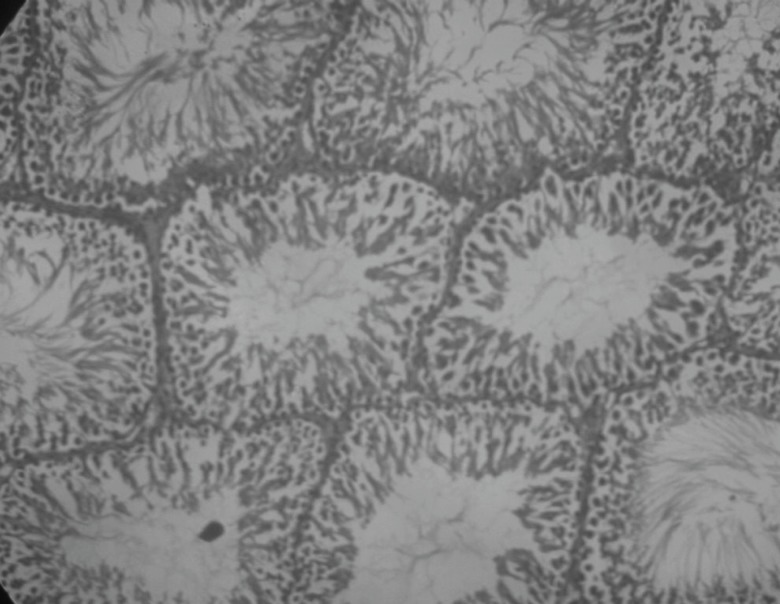
Histological picture of rats’ testis after treating with insulin and rutin

**Figure 12:**
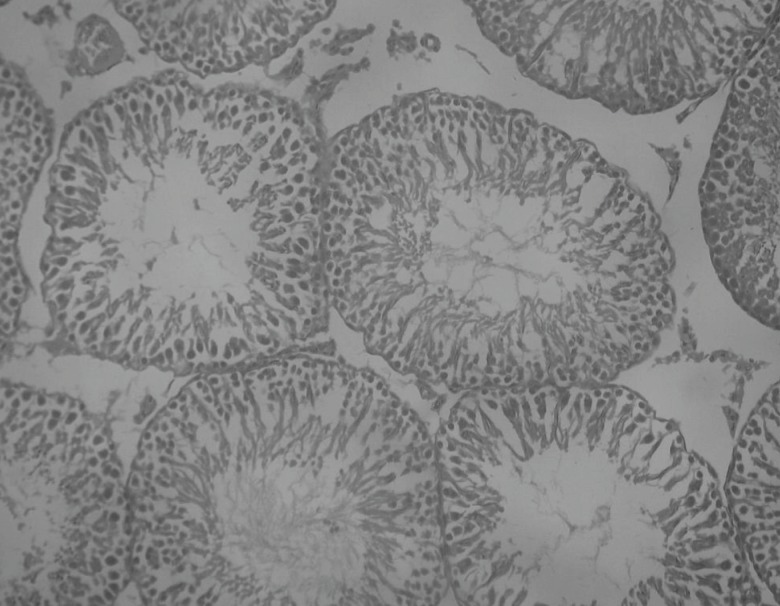
Histological picture of rats’ testis after treating with insulin and Naringin

Insulin treated rats, Rutin alone treated rats and Naringin alone treated rats have shown improvement in testicular histology. Rats which have been treated only on insulin have shown maximum degree of protection against diabetes induced disturbance in testicular architecture. Diabetic animals treated with insulin in combination with rutin and insulin in combination with Naringin, have shown almost normal testicular architecture as comparable with normal control rats (Figures 11 and 12).

## Discussion and Conclusion

Diabetes mellitus (DM) is the most common metabolic disorder. Type 1 DM is associated with many complications including male infertility. It is more likely that the long-term and uncontrolled DM with sustained high blood glucose levels causes oxidative stress (27). Increased lipid peroxidation and the increased imbalance causes impairment of the steroidogenic function of the testis. Lipid peroxidation is an important biological consequence of oxidative cellular damage in patients with DM. Lipoperoxidation products such as malondialdehyde (MDA) reflects oxidative stress. Cellular and tissue damage is thought to be an important factor in the pathogenesis of DM and its complications (28). Studies have detected increased semen ROS levels in 25% to 40% of infertile men (29, 30). 

Experimentally, streptozotocin (STZ) induces DM, probably through the generation of ROS, leading to islet cell destruction (31). The balance between oxidant and antioxidant species has been proposed to have an important role in preventing diabetic complications. Dietary antioxidants play a major role in the maintenance of the oxidative balance. Bioflavonoids considered as efficacious antioxidants. Rutin and Naringin belong to the class of bioflavonoids widely distributed in fruits and vegetables and reported for their antioxidant and anti-diabetic activity (32). Rutin and Naringin were tested for their *in-vivo *and *in-vitro *antioxidant activity in our laboratory. They were proved as cardioprotective, nephroprotective and cerebroprotective in different animal models (33). In the current study, Rutin and Naringin were evaluated for their antioxidant and sperm protectant activities in type 1 diabetes mellitus animals. 

In this study, streptozotocin treated rats showed high levels of blood glucose and insulin effectively normalized the glucose levels. Rutin and Naringin at 10 mg/kg/day had no effect on blood glucose level of streptozotocin induced diabetic rats. But Rutin and Naringin combined with insulin showed effective control of blood glucose compared to rats treated with only insulin. Previous study reported that glycosylated flavonoids and their complexes with vanadium have antihyperglycemic effect in alloxan-induced diabetic rats (34). In another study, oral administration of rutin (100 mg/kg) to diabetic rats for a period of 45 days resulted in significant decrease in plasma glucose and increase in insulin levels (35). But in another previous study, Naringin 10 mg/kg has not shown any improvement in blood glucose levels (32). This indicates that the intra-peritoneal administration of Rutin and Naringin shows anti-hyperglycemic activity in high doses. Above reports suggested that bioflavonoids had shown anti-diabetic activity only at high dose level. In our study, we used 10 mg/kg of rutin and naringin and found that they had no effect on blood glucose levels. These results are in accordance with the previous reports.

The present study demonstrates that diabetic control group had shown significant reduction in sperm parameters like sperm motility, sperm count and sperm viability, in comparison with normal rats. Earlier works had reported that type 1 Diabetes mellitus had deleterious effects on male reproductive system. Type I diabetes mellitus may affect endocrine function and spermatogenesis (36, 37). In diabetes, mellitus hyperglycemia increases oxidative stress (ROS) and it causes DNA damage in all tissues like retina, renal, brain, myocardium and testis (2). Moreover, increased lipid peroxidation and decreased antioxidant enzyme concentration mainly impairs male fertility in type 1 Diabetes mellitus (13). This increased oxidative stress in diabetes mellitus attacks polyunsaturated fatty acids (PUFA) in the sperm cell membrane and results in lipid peroxidation. This increased lipid peroxidation causes DNA damage in sperm cell (3). Therefore, it was suggested that oxidative stress plays a major role in male infertility associated with diabetes mellitus. 

In the present study, bioflavonoids, like Rutin and Naringin, had shown significant stimulating effects on sperm parameters like sperm motility, sperm count and sperm viability administered along with insulin, compared to insulin treated group. These results are in agreement with the previous studies of bioflavonoids effect on male reproductive system. Bioflavonoid quercetin shows effect on the function of prostate by interacting with prostatic type II sites (38). Previous study of N.R. Desroches *et al*., in 2005, demonstrated that Blueberry leaf extract and quercetin reduces the lipid peroxidation and improves capacitation of spermatozoa in *in-vitro *study (29). They suggested that the flavonoids quercetin improve sperm motility of rat sperm after 3-4 h incubation. Antioxidants, like vitamin E, had shown improved steroidogenesis and ROS in diabetic rat testis (13). Some anti diabetic plants like aqueous extract of G. procumbens restores the diabetes induced sperm damage (1). Rutin and Naringin along with insulin treated groups shows stimulating effects on all sperm parameters like sperm motility, sperm count and sperm viability, in type I diabetic rats. Rutin with the dose of 10 mg/kg along with insulin offers more stimulating effects on all sperm parameters in comparison with all other groups. 

In our study, testicular MDA levels were elevated and in diabetic control group animals and the antioxidant parameters were reduced (SOD and Catalase). Oxidative stress-mediated damage to the sperm plasma membrane, integrity of DNA and on germ cell leads to deterioration of sperm quality (1, 3). With the administration of insulin, Rutin and Naringin alone or in combination, MDA levels were effectively reduced and the SOD increased; Catalase levels comparable with the corresponding normal control value were also achieved. These results suggest that hyperglycemia accompanied by increased free radical formation causes a reduction in endogenous antioxidant capacity, leading to enhanced oxidative stress. Several studies reported the effectiveness of Rutin and Naringin as antioxidants. Rutin reduces the lipid peroxidation in *in-vitro *method (40). Previous reports suggested that Rutin has anti-lipoperoxidation activity by scavenging hydroxyl and superoxide radicals (41). Jeon s.m *et al*. (2002), suggested that Naringin reduces the lipid peroxidation in cholesterol fed animals by scavenging free radicals that are generated. (42) Ali MM, El Kader MA *et al*., (2004) reported that naringin reduces the oxidative stress in diabetes mellitus by scavenging free radicals generated by hyperglycemia (43, 44). In 2006, Jungsook Cho *et al*.,proved that, flavonoid hesperidin protects brain by scavenging free radicals (45, 46). 

There are several reports describing elevations in specific oxidant stress markers in both experimental STZ and human diabetes mellitus, together with reduced total antioxidant defense and depletion of individual antioxidants (47). Increased prooxidant levels increase lipid peroxidation products and hydrogen peroxide in diabetes mellitus. This prooxidants inhibit the activity of antioxidative enzymes CAT, SOD, and GPX as well as total antioxidant status (48). 

In our study, rutin has shown better effect in comparison with naringin. Rutin belongs to the class of flavonols and naringin belongs to flavonones. It is well noted and proved in several studies that flavonols are much active in delivering therapeutic benefit, compared to flavonones. (49)

In conclusion, both rutin and naringin in combination with insulin restored normal testicular function including sperm parameters, SOD and catalase levels reduction in MDA levels. The probable mechanism of the action of rutin and naringin might be by decreasing the lipid peroxidation and by increasing the levels of antioxidant enzymes. 
